# Efficacy of Tislelizumab plus Lenvatinib in hepatocellular carcinoma after curative hepatectomy: a real-world study

**DOI:** 10.3389/fimmu.2025.1652717

**Published:** 2025-09-18

**Authors:** Kai Chen, Xu Feng, Yuan Shi, Heng Xiao, Xiang Lan, Zheng-Rong Shi

**Affiliations:** Department of Hepatobiliary Surgery, The First Hospital of Chongqing Medical University, Chongqing, China

**Keywords:** hepatocellular carcinoma, adjuvant therapy, tislelizumab, Lenvatinib, real-world study

## Abstract

**Background:**

The efficacy of adjuvant therapy after curative resection for hepatocellular carcinoma (HCC) patients is still debated. This study aimed to evaluate the impact of adjuvant Tislelizumab combined with Lenvatinib on the prognosis of patients with HCC after hepatectomy.

**Methods:**

Patients diagnosed with HCC and undergoing curative hepatectomy were retrospectively enrolled, and stratified into two groups: the Hepatectomy alone group and the Hepatectomy−T-L group, based on whether they received adjuvant therapy with Tislelizumab and Lenvatinib. The primary endpoint was disease-free survival (DFS); the secondary endpoints included overall survival (OS) and adverse events.

**Results:**

A total of 288 patients were enrolled and assigned to the Hepatectomy alone group (n=256) and the Hepatectomy−T-L group (n=32) between January 2019 and December 2023. Baseline demographic and clinical characteristics were well balanced between the two groups. The median follow−up time was 28.73 months (95% CI 26.03–31.43 months). The median DFS was significantly longer in the Hepatectomy-T-L group than in the Hepatectomy Alone group [40.78 months (95% CI 29.25–52.31) vs. 28.80 months (95% CI 25.52–32.08), hazard ratio [HR] = 0.51 (95% CI 0.28–0.91), P = 0.021]. The median OS was also significantly longer in the Hepatectomy-T-L group than in the Hepatectomy Alone group [42.10 months (95% CI 37.55–46.65) vs. 34.00 months (95% CI 30.40–37.60), HR = 0.36 (95% CI 0.18–0.70), P = 0.0018]. Adverse events were more frequently observed in the Hepatectomy-T-L group. The incidence of adverse events (AEs) was compared and manageable between the two groups.

**Conclusions:**

Adjuvant Tislelizumab and Lenvatinib after curative hepatectomy holds significant potential benefits with manageable adverse events.

## 1 Introduction

Hepatocellular carcinoma (HCC) ranks as the sixth most prevalent malignancy worldwide and the fourth in tumor-related mortality, with a significant global health burden (1). Despite advances in medical technology, the prognosis for HCC patients continues to be poor due to high recurrence rates following surgical intervention. Several studies have indicated that approximately 50-70% of patients suffered from recurrence within five years after hepatectomy (2–4). Curative hepatectomy, the standard treatment for resectable HCC (5), often fails to prevent recurrence, necessitating the exploration of effective adjuvant therapies.

Adjuvant treatments aim to reduce the risk of recurrence and improve overall survival rates (6). In recent years, immunotherapy and targeted therapy have gained significant attention for their potential benefits in HCC management (7–10). In a global, open-label phase 3 study (IMbrave050), improved recurrence-free survival was observed in high-risk hepatocellular carcinoma patients who received atezolizumab plus bevacizumab following curative resection or ablation, compared with those who underwent active surveillance (11). However, previous trials assessing single-agent adjuvant therapy have yielded negative or inconclusive results. The STORM study, which evaluated sorafenib as adjuvant therapy after resection or ablation, failed to show improvement in recurrence-free survival (RFS) or overall survival (OS) compared to placebo (12). Similarly, the CheckMate 9DX and KEYNOTE-937 phase III trials are ongoing to assess the role of adjuvant PD-1 monotherapy (nivolumab and pembrolizumab, respectively), but results are not yet available. These trials illustrate the limitations of monotherapy approaches and underscore the need to explore more effective combination strategies.

Another multicenter phase III randomized controlled trial (RCT) indicated that FOLFOX-based hepatic arterial infusion chemotherapy (FOLFOX-HAIC) significantly lowered the recurrence risk and improved survival in hepatocellular carcinoma patients with microvascular invasion (MVI). The median disease-free survival (DFS) was 20.3 months (95% CI, 10.4 to 30.3) in the treatment group versus 10.0 months (95% CI, 6.8 to 13.2) in the control group (hazard ratio, 0.59; 95% CI, 0.43 to 0.81; P = 0.001) (13). Collectively, these findings highlight the promising role of adjuvant therapies in enhancing outcomes after curative hepatectomy for HCC. Yet, a globally accepted standard adjuvant treatment protocol remains elusive.

Beyond clinical efficacy, the evolving role of cancer immuno-oncology (IO) in HCC has highlighted the importance of identifying predictive biomarkers and characterizing the tumor immune microenvironment (TIME) to optimize therapeutic benefit. Importantly, TIME fundamentally shapes the expression and prognostic relevance of these biomarkers, thereby linking immune contexture with therapeutic responsiveness. Features such as PD-L1 expression, α-fetoprotein (AFP), immune gene signatures, and tumor-infiltrating lymphocytes have been studied for their potential to predict responsiveness to immunotherapy (14–16). Among these, PD-L1 expression has been investigated as a key biomarker, with higher expression levels generally correlating with improved response to checkpoint inhibitors, though its predictive value remains inconsistent due to heterogeneity in detection methods and tumor biology (17, 18). Similarly, early dynamic changes in AFP levels during treatment have shown promise as a non-invasive predictor of treatment response and survival outcomes, providing practical utility in clinical monitoring despite its limitations in AFP-negative patients (19, 20). Additionally, the presence of an “immune-excluded” or “immune-desert” microenvironment in HCC has been associated with resistance to immune checkpoint blockade, suggesting that personalized approaches may be necessary (21–23). These advances support the growing need to integrate immune landscape characterization into treatment planning. Furthermore, novel immunotherapeutic strategies including adoptive T-cell therapies and oncolytic virotherapy are under investigation, expanding the horizon of immune-oncology (IO) in HCC (24–26). These emerging modalities may eventually be combined with existing checkpoint inhibitors or targeted therapies to improve efficacy.

Given the limitations of existing adjuvant therapies, novel combinations such as Tislelizumab and Lenvatinib are being explored for their potential to address the high recurrence rates post-surgery. Tislelizumab, an immune checkpoint inhibitor targeting PD-1, has shown promise in enhancing the immune response against tumor cells (27, 28). Its efficacy and non-inferiority to sorafenib in advanced HCC have been demonstrated in the RATIONAL 301 phase III trial, which showed comparable overall survival and a more favorable safety profile, supporting its potential for broader application in HCC management (29). Concurrently, Lenvatinib, an inhibitor of multiple receptor tyrosine kinases, has demonstrated efficacy in inhibiting tumor angiogenesis and proliferation (30–32). The pivotal REFLECT study established Lenvatinib as a non-inferior alternative to sorafenib in first-line treatment of unresectable HCC, with superior progression-free survival and objective response rate (33). The synergistic effect of these agents could potentially offer a robust strategy to prevent HCC recurrence after surgery.

Inspired by the success of the IMbrave050 regimen and the biological rationale for combining immune checkpoint blockade with anti-angiogenic therapy, the combination of Tislelizumab plus Lenvatinib may offer a similarly synergistic strategy in the adjuvant setting. This study aims to assess the prognostic impact of Tislelizumab plus Lenvatinib as adjuvant therapy, providing critical insights into their potential to improve disease-free survival and overall survival in HCC patients following curative hepatectomy. The results of such studies could redefine the therapeutic landscape for HCC and offer new hope for patients undergoing curative hepatectomy.

## 2 Materials and methods

### 2.1 Study population

All consecutive 307 patients were retrospectively reviewed from January 2019 to December 2023 from a tertiary hospital in Chongqing, China. Patients with histologically confirmed HCC who underwent curative hepatectomy were included. As a retrospective real-world study, the sample size was not predetermined but was based on the total number of patients who met the eligibility criteria and received adjuvant tislelizumab plus lenvatinib during the study period. No formal sample size calculation was conducted.

Eligibility criteria included: (1) Histologically confirmed HCC; (2) Age between 18 and 75 years; (3) Child-Pugh class A or B liver function; (4) No evidence of extrahepatic metastasis; (5) Eastern Cooperative Oncology Group (ECOG) performance status of 0–1; (6) Adequate hematologic and renal function as indicated by routine laboratory assessments. Patients were excluded if they: (1) had received any prior systemic therapy, including targeted therapies or immunotherapies; (2) had a history of other malignancies within the last five years; (3) had significant comorbidities such as uncontrolled hypertension, heart failure, or renal insufficiency; (4) were unable or unwilling to provide informed consent; or (5) experienced recurrence or death within 30 days postoperatively.

### 2.2 Hepatectomy and adjuvant therapy

All patients scheduled for hepatectomy were subject to preoperative review by a multidisciplinary team. Surgical techniques included both laparoscopic and open surgery, and all resections were carried out by senior surgeons. After mobilization of the liver, intraoperative ultrasound was employed to precisely locate the tumor and detect possible intrahepatic metastases. Based on tumor’s location, either an anatomical or non-anatomical resection was performed. The liver parenchyma was dissected using a harmonic scalpel and Cavitron Ultrasonic Surgical Aspirator (CUSA) to minimize blood loss and tissue damage. The Pringle maneuver was applied to control the inflow of liver.

Adjuvant therapy was initiated one month after surgery. All patients received intravenous tislelizumab at a dose of 200 mg on day 1 of a 21-day cycle, in combination with lenvatinib administered orally at 12 mg daily for patients weighing ≥60 kg, or 8 mg for those weighing <60 kg (34). This dosing protocol was informed by a phase 2 study that showed the combination of tislelizumab and lenvatinib to have a manageable safety profile, adhering to the prescribing information for each drug (35). In cases where patients experienced severe, intolerable adverse events, treatment was discontinued.

### 2.3 Data collection and follow-up

The clinical data for all eligible patients were retrospectively extracted from the medical records systems of each participating hospital. The following parameters were collected: age, sex, ECOG performance status, hepatitis B virus (HBV) status, cirrhosis, Child-Pugh grade, preoperative serum alpha-fetoprotein (AFP) levels, maximum tumor size, number of tumors, tumor differentiation, postoperative pathological findings of microvascular invasion (MVI), presence of major vascular invasion, poor tumor differentiation, disease-free survival (DFS), and overall survival (OS).

Postoperatively, patients were followed up every two months for the initial two years, transitioning to follow-ups every three to six months thereafter. During these visits, serum tumor markers were assessed, and abdominal ultrasounds were conducted. To detect metastasis or early recurrence, contrast-enhanced computed tomography (CT) or magnetic resonance imaging (MRI) scans were performed every three months. Should any indications of tumor recurrence or metastasis arise, additional diagnostic and treatment measures would be initiated based on the results. Adverse events (AEs) were recorded and evaluated according to the National Cancer Institute Common Terminology Criteria for Adverse Events (version 5.0) (36).

### 2.4 Statistical analyses

Categorical variables were expressed as counts (n, %) and compared using the chi-square test or Fisher’s exact test appropriately. Continuous variables were categorized as necessary, based on clinical relevance. Kaplan-Meier curves were utilized to evaluate cumulative survival, with comparisons made through the log-rank test. Both univariable and multivariable Cox regression analyses were conducted to identify independent prognostic factors associated with DFS and OS in patients with HCC following curative hepatectomy. Variables with p < 0.1 in univariable analyses were included in the multivariable Cox models. P < 0.05 was considered statistically significant. All statistical analyses were performed with the Statistical Package for the Social Sciences (SPSS) version 27 and R version 4.4.0.

## 3 Results

### 3.1 Patient characteristics

**Figure 1:**
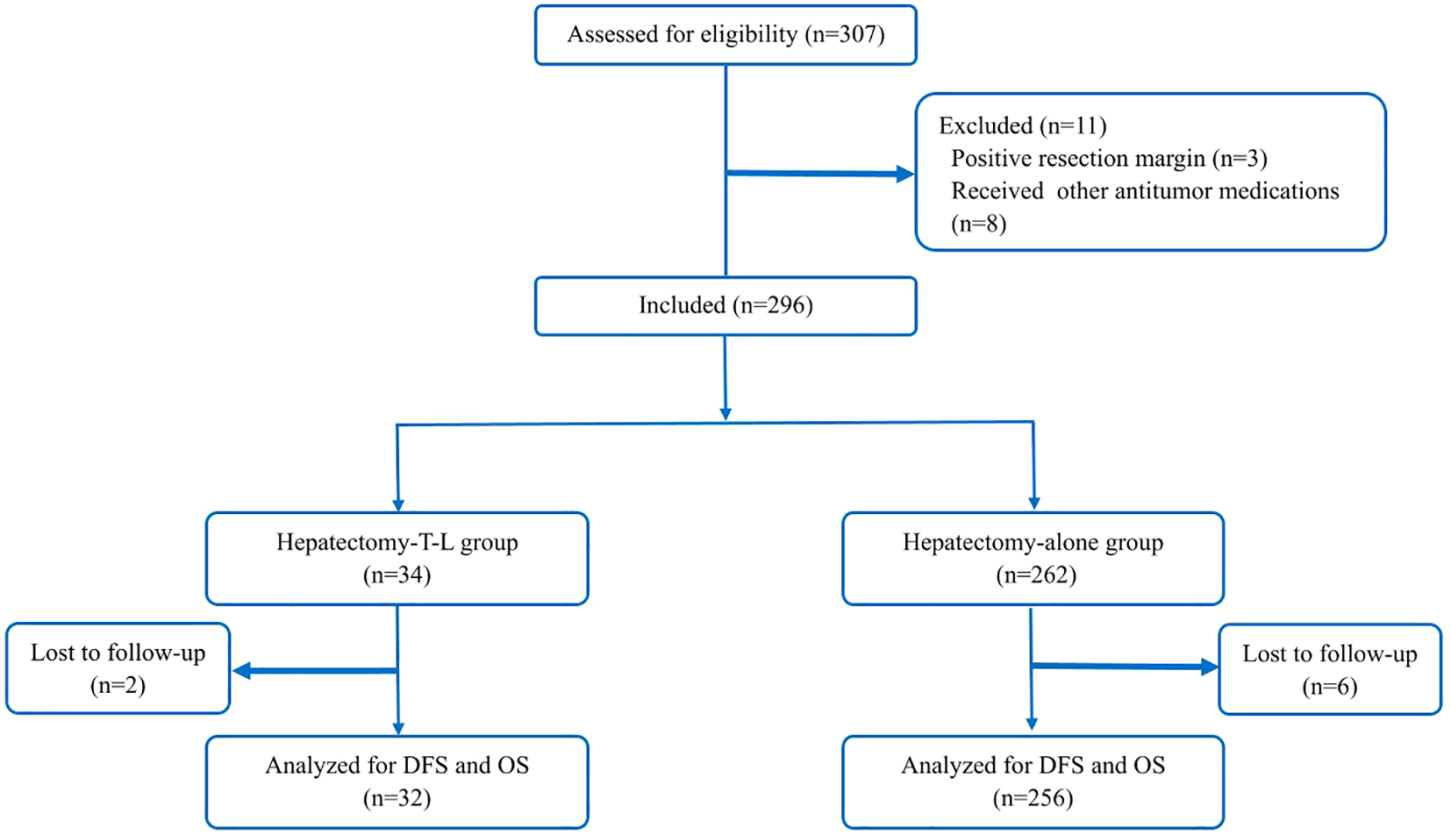
A flow diagram illustrating the overall patient enrollment.

A total of 296 patients were included in the study and subsequently categorized into two groups: those receiving adjuvant therapy with Tislelizumab and Lenvatinib following curative hepatectomy (the Hepatectomy-T-L group, n = 34) and those undergoing hepatectomy alone (the Hepatectomy-alone group, n = 262). In the Hepatectomy-T-L group, 2 patients were lost to follow-up, resulting in their exclusion from the analysis. Similarly, in the Hepatectomy alone group, 6 patients were lost to follow-up and were excluded from the final analysis. Consequently, a total of 288 patients were included in the final analysis: 32 in the Hepatectomy-T-L group and 256 in the Hepatectomy-alone group (**Figure 1**). Baseline demographic and clinical characteristics were well balanced between the two groups (**Table 1**).

**Table 1:** Baseline characteristics of the two group patients.

Characteristic	Hepatectomy−t-l group(n=32)	Hepatectomy alone group(n=256)	P
Age(n, %)			
<65y	26(81.3%)	172(67.2%)	0.11
≥65y	6(18.7%)	84(32.8%)	
Sex (n,%)			
Male	29(90.6%)	224(87.5%)	0.61
Female	3(9.4%)	32(12.5%)	
ECOG performance status(n, %)			
0	29(90.6%)	238(93%)	0.87
1	2(6.3%)	13(5%)	
2	1(3.1%)	5(2%)	
HBV(n,%)			
Present	25(78.1%)	184(71.9%)	0.45
Absent	7(21.9%)	72(28.1%)	
Cirrhosis(n, %)			
Present	21(65.6%)	182(71.1%)	0.52
Absent	11(34.4%)	74(28.9%)	
Child–Pugh grade(n, %)			
Class A	30(93.8%)	238(93%)	0.87
Class B	2(6.2%)	18(7%)	
Preoperative serum AFP(n, %)			
≥ 400 ng/L	10(31.3%)	67(26.2%)	0.54
< 400 ng/L	22(68.8%)	189(73.8%)	
Maximum tumor size(n, %)			
>5 cm	11(34.4%)	95(37.1%)	0.76
≤ 5 cm	21(65.6%)	161(62.9%)	
Multiple tumors (n, %)			
≥ 3	0(0%)	10(3.9%)	0.61
< 3	32(100%)	246(96.1%)	
Microvascular invasion(n, %)			
0	17(53.1%)	145(56.6%)	0.41
1	14(43.8%)	89(34.8%)	
2	1(3.1%)	22(8.6%)	
Macrovascular invasion(n, %)			
Present	2(6.3%)	19(7.4%)	0.81
Absent	30(93.7%)	237(92.6%)	
Poor tumor differentiation(n, %)			
Present	7(21.9%)	57(22.3%)	0.96
Absent	25(78.1%)	199(77.7%)	

### 3.2 Overall survival and disease-free survival

After a median follow-up period of 28.73 months (95% CI, 26.03-31.43 months), 126 (43.7%) patients had tumor recurrence (113 in the Hepatectomy-alone group and 13 in the Hepatectomy-T-L group), and 104 (36.1%) patients died (94 in the Hepatectomy-alone group and 10 in the Hepatectomy-T-L group).

**Figure 2:**
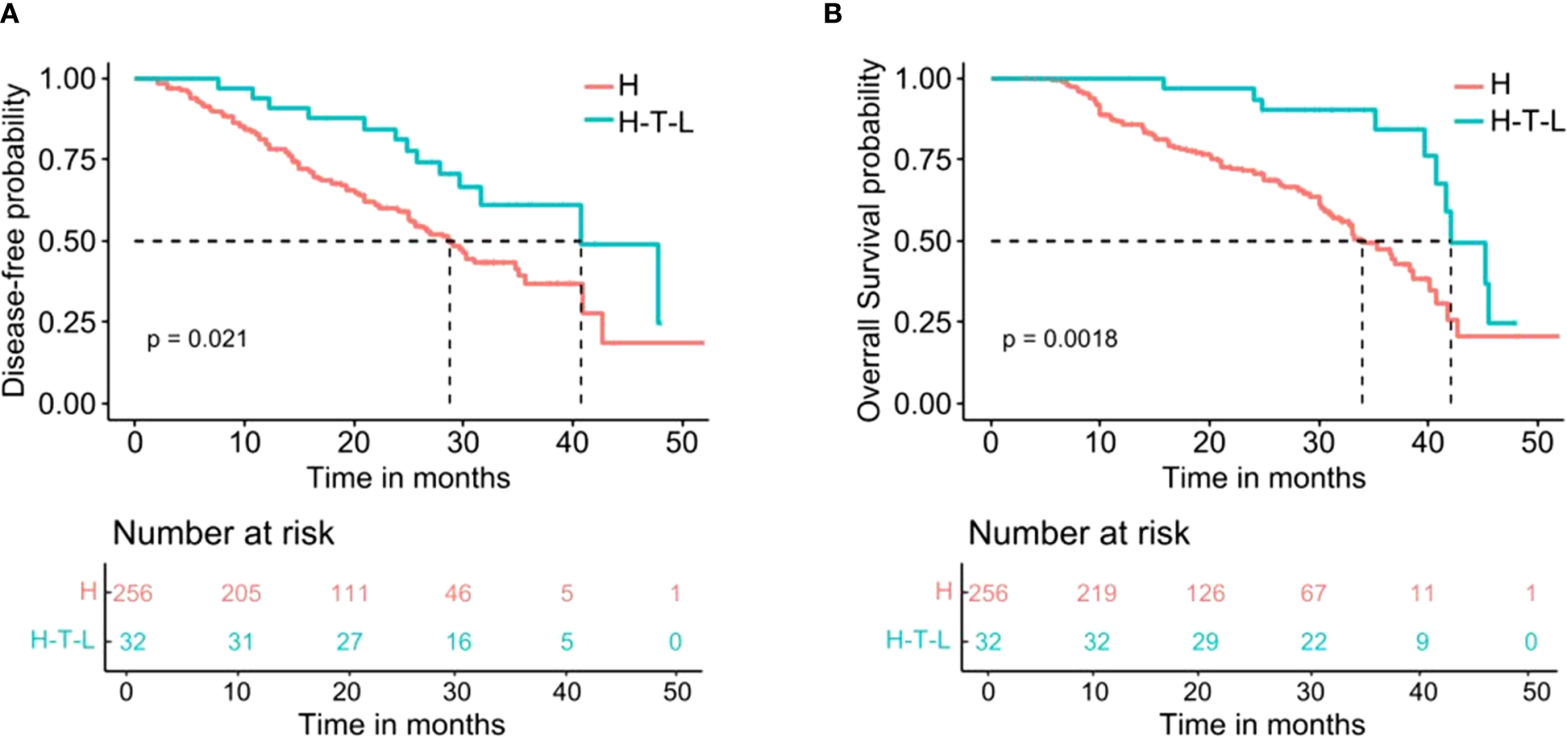
Kaplan-Meier analysis for survival in patients with HCC after hepatectomy stratified by adjuvant tislelizumab and lenvatinib. **(A)** Disease-free survival. **(B)** Overall survival. H indicates the Hepatectomy alone group; H-T-L means the Hepatectomy-T-L group.

The median DFS was 40.78 months (95% CI 29.25–52.31) in the Hepatectomy-T-L group and was 28.80 months (95% CI 25.52–32.08) in the Hepatectomy Alone group (HR = 0.51, 95% CI 0.28–0.91, P= 0.021; **Figure 2A**). The 1-, 2-, and 3-year DFS rates for the Hepatectomy-T-L group were 90.6%, 81.0%, and 61.2%, and were 79.6%, 59.5%, and 36.9% for the Hepatectomy Alone group, respectively.

The median OS for the Hepatectomy-T-L group was 42.10 months (95% CI 37.55–46.65) and was 34.00 months (95% CI 30.40–37.60) in the Hepatectomy Alone group (HR = 0.36, 95% CI 0.18–0.70, P= 0.0018; **Figure 2B**). The 1-, 2-, and 3-year OS rates for the Hepatectomy-T-L group were 96.8%, 90.0%, and 75.9% and were 85.9%, 70.8%, and 45.9% for the Hepatectomy Alone group, respectively.

### 3.3 Univariable and multivariable cox regression analysis

Univariable and multivariable cox regression analysis were performed to assess the association between various clinical and pathological factors with OS and DFS. In the univariable analysis, we identified that HBV infection, macrovascular invasion, and postoperative adjuvant therapy were significantly associated with both OS and DFS. Multivariable analysis further revealed that HBV infection (HR: 2.72, 95% CI 1.575–4.699, P = 0.001), macrovascular invasion (HR: 2.824, 95% CI 1.433–5.565, P = 0.003), and postoperative therapy (HR: 0.333, 95% CI 0.167–0.664, P = 0.024) were independent risk factors for OS. Similarly, HBV infection (HR: 2.126, 95% CI 1.333–3.391, P = 0.002), macrovascular invasion (HR: 2.577, 95% CI 1.380–4.815, P = 0.003), and postoperative therapy (HR: 0.51, 95% CI 0.282–0.921, P = 0.026) were found to be significantly associated with DFS (**Table 2**).

**Table 2:** Univariable and multivariable Cox regression analyses of risk factor with overall survival and disease-free survival.

Factors	Overall survival				Disease-free survival			
	Univariate analysis p value	Multivariate analysis HR	95%CI	P value	Univariate analysis p value	Multivariate analysis HR	95%CI	P value
Age	0.41				0.122			
<65y								
≥65y								
Sex	0.14				0.819			
Female								
Male								
ECOG	0.582				0.99			
0								
1								
2								
HBV	0.003	2.72	1.575,4.699	0.001	0.003	2.126	1.333,3.391	0.002
Absence								
Presence								
Cirrhosis	0.224				0.51			
Absence								
Presence								
Child–Pugh grade(n, %)	0.984				0.586			
Class A								
Class B								
AFP	0.814				0.839			
<400 ng/mL								
≥400 ng/mL								
Maximum tumor size	0.464				0.642			
≤5cm								
>5cm								
Tumor number	0.245				0.484			
<3								
≥3								
MVI	0.128				0.165			
0								
1								
2								
Macrovascular invasion	0.008	2.824	1.433,5.565	0.003	0.003	2.577	1.380,4.815	0.003
Absence								
Presence								
Poor tumor differentiation(n, %)	0.354				0.911			
Absence								
Presence								
Postoperative therapy	0.003	0.333	0.167,0.664	0.002	0.024	0.51	0.282,0.921	0.026
Absence								
Presence								

### 3.4 Adverse events

The incidence of adverse events (AEs) was compared between the Hepatectomy-Alone and Hepatectomy-T-L groups (**Table 3**). Common AEs included abdominal pain (40.6% vs. 26.2%), elevated ALT (50.0% vs. 40.2%), and anemia (37.5% vs. 29.7%), with no significant differences between the groups (p > 0.05). Notably, hyperbilirubinemia was more frequent in the Hepatectomy-T-L group (31.3% vs. 16.8%, p = 0.047). There were also higher rates of hypertension (31.3% vs. 18.0%, p = 0.073) and leukopenia (34.4% vs. 20.7%, p = 0.079) in the Hepatectomy-T-L group, although these differences did not reach statistical significance. For grade 3/4 AEs, hypertension was significantly more prevalent in the Hepatectomy-T-L group, with 2 cases (6.2%) compared to none in the Hepatectomy-Alone group (p < 0.001). Fever (3.1% vs. 3.2%) and hyperbilirubinemia (3.1% vs. 2.3%) were similarly distributed between the two groups (p > 0.05).

**Table 3:** Adverse events.

Adverse events	Any grade			Grade 3/4		
	Hepatectomy alone (n = 256)	Hepatectomy-t-l (n = 32)	P value	Hepatectomy alone (n = 256)	Hepatectomy-t-l (n = 32)	P value
Hypertension	46(18.0%)	10(31.3%)	0.073	0	2 (6.2%)	<0.001
Diarrhea	53(20.7%)	7(21.9%)	0.878	0	0	1
Vomiting	52(20.3%)	10(31.3%)	0.156	0	0	1
Inappetence	68(26.6%)	8(25.0%)	0.85	0	0	1
Abdominal pain	67(26.2%)	13(40.6%)	0.085	0	0	1
Fever	32(12.5%)	6(18.8%)	0.325	4(3.2%)	1(3.1%)	0.359
Anemia	76(29.7%)	12(37.5%)	0.366	0	0	1
Leukopenia	53(20.7%)	11(34.4%)	0.079	0	0	1
Elevated ALT	103(40.2%)	16(50.0%)	0.29	0	0	1
Elevated AST	81(31.6%)	10(31.3%)	0.964	0	0	1
Hypoalbuminemia	42(16.4%)	7(21.9%)	0.438	0	0	1
Hyperbilirubinemia	43(16.8%)	10(31.3%)	0.047	6(2.3%)	1(3.1%)	0.787

## 4 Discussion

Hepatectomy remains a cornerstone curative treatment for hepatocellular carcinoma, offering the potential for long-term survival (4). Nevertheless, postoperative recurrence continues to pose a major clinical challenge, significantly compromising long-term outcomes (37, 38). Given the limited therapeutic options available once recurrence occurs, preventing postoperative relapse is of paramount importance. While adjuvant therapies have shown promise in reducing recurrence and improving survival, no consensus exists regarding the optimal postoperative regimen for HCC (39). This underscores an urgent unmet need in clinical practice. Against this backdrop, our study investigated the prognostic effect of adjuvant tislelizumab combined with lenvatinib following curative hepatectomy, aiming to provide real-world insights into this critical area of clinical uncertainty.

Our study demonstrated that adjuvant tislelizumab plus lenvatinib significantly prolonged median DFS (40.78 months) and OS (42.10 months) in patients undergoing curative hepatectomy. These findings align with previous studies exploring adjuvant therapies in HCC. A randomized trial reported that adjuvant transarterial chemoembolization (TACE) significantly improved median DFS (17.45 months vs. 9.27 months, HR = 0.70, P = 0.020) and OS (44.29 months vs. 22.37 months, HR = 0.68, P = 0.029) in patients with single tumors >5 cm and MVI (40). Similarly, a phase III clinical trial demonstrated that FOLFOX-HAIC significantly prolonged DFS in MVI-positive patients (20.3 months vs. 10.0 months, HR = 0.59, P = 0.001), although no significant OS benefit was observed (13). Notably, while TACE and HAIC have shown survival benefits, they are invasive procedures associated with additional procedural risks and complications. In contrast, the combination of targeted therapy and immunotherapy in this study not only achieved survival outcomes comparable to or even exceeding those reported with postoperative TACE/HAIC but also minimized procedural invasiveness, potentially improving patient compliance and tolerability. These findings highlight the promise of immunotherapy-based strategies as a non-invasive yet effective approach for improving long-term outcomes in postoperative HCC patients.

In the present study, HBV infection, macrovascular invasion, and postoperative adjuvant therapy emerged as independent prognostic factors influencing both disease recurrence and overall survival. The prognostic impact of HBV infection and macrovascular invasion has been well documented, as both factors are closely linked to tumor aggressiveness, increased recurrence rates, and poor long-term outcomes in patients undergoing hepatic resection for HCC (41–43). In contrast, the role of postoperative adjuvant therapy, particularly systemic therapy involving targeted agents and immune checkpoint inhibitors, remains less clearly defined. Notably, the IMbrave050 trial marked a breakthrough by establishing the potential of atezolizumab plus bevacizumab as an adjuvant regimen following resection or ablation. While this study confirmed a significant benefit in recurrence-free survival, it did not yet provide mature data on overall survival (11). Similarly, a recent multicenter phase 2 randomized trial in China evaluated adjuvant sintilimab in HCC patients with microvascular invasion and demonstrated a significant improvement in recurrence-free survival compared to active surveillance (27.7 vs. 15.5 months; HR = 0.534; P = 0.002), though OS data remain immature (10). Compared to our findings, which showed longer DFS and OS with combination therapy, the differences may stem from the limited efficacy of PD-1 monotherapy, shorter treatment duration (eight cycles of sintilimab), and relatively brief follow-up. Furthermore, several large-scale randomized trials are currently ongoing to evaluate various immune checkpoint inhibitors in the adjuvant setting, including CheckMate 9DX (nivolumab), EMERALD-2 (durvalumab plus bevacizumab), and KEYNOTE-937 (pembrolizumab). These studies are expected to provide more definitive guidance regarding optimal regimens, patient selection, and long-term survival benefit.

It is worth noting that most existing studies on adjuvant therapy have focused on patients with high-risk features, particularly those with MVI. However, in real-world clinical practice, the application of adjuvant systemic therapy has expanded to include selected patients without MVI based on individualized risk assessments. In our study, the proportion of MVI-positive patients was similar between groups, yet those receiving adjuvant tislelizumab plus lenvatinib exhibited significantly improved survival. This observation suggests that even patients lacking classical high-risk features may derive benefit from postoperative systemic therapy. One possible explanation is that other unfavorable pathological or intraoperative factors—such as poor differentiation, satellite nodules, or close surgical margins—may have influenced treatment decisions.

The current findings hold substantial clinical potential, especially for patients not eligible for locoregional adjuvant therapies or those intolerant to intensive chemotherapy. The ability to administer systemic therapy with manageable toxicity widens the scope of adjuvant treatment in clinical practice. Looking forward, key knowledge gaps include identifying which subpopulations derive the most benefit from immuno-targeted adjuvant therapy, determining optimal treatment durations, and integrating biomarker-driven patient selection. We anticipate that within the next five years, ongoing RCTs and real-world studies will refine clinical guidelines, possibly leading to the adoption of immuno-targeted combinations as standard postoperative strategies for selected HCC patients.

Regarding treatment-related adverse events (AEs), the overall incidence of AEs was comparable between the two groups. However, patients in the adjuvant therapy group experienced a higher frequency of hyperbilirubinemia and hypertension. Notably, grade 3/4 hypertension occurred more frequently in this group, though no AE-related deaths were reported. These findings suggest that while certain toxicities, particularly hypertension, may be more common with postoperative immuno-targeted therapy, the safety profile remains generally manageable. With appropriate monitoring and supportive care, most AEs were controllable and did not compromise the continuation of treatment.

As a retrospective study, our research inevitably carries certain limitations, including potential selection bias and limited control over confounding variables. Moreover, the relatively small sample size may restrict the generalizability of the findings. While propensity score matching was attempted, the resulting sample size was insufficient for robust statistical analysis. Nevertheless, our study offers a novel perspective: in the current era where immunotherapy and targeted therapy are increasingly prevalent, postoperative adjuvant therapy may confer additional survival benefits without substantially increasing postoperative complications. More importantly, the potential benefit observed even in patients beyond classical high-risk categories highlights a possible paradigm shift toward broader use of systemic immuno-targeted strategies in the adjuvant setting. Future large-scale prospective trials are essential to confirm these findings and refine patient selection criteria.

## 5 Conclusion

Adjuvant Tislelizumab and Lenvatinib after curative hepatectomy holds significant potential benefits with manageable adverse events.

## Data Availability

The original contributions presented in the study are included in the article/supplementary material. Further inquiries can be directed to the corresponding authors.
